# Long-term toxicological studies on the Chinese medicine 2036 Specialty-Qiangxin recipe in rats

**DOI:** 10.1080/13880209.2021.1967410

**Published:** 2021-08-31

**Authors:** Andong Zhao, Yi Yang, Xiaohua Pan, Manhon Chung, Sa Cai, Yu Pan

**Affiliations:** a Health Science Center, Shenzhen University, Shenzhen, China; b Department of Trauma and Orthopedics, The 2nd Affiliated Hospital of Shenzhen University, The Affiliated Baoan Hospital of Southern Medical University, Shenzhen, China; c Department of Plastic and Reconstructive Surgery, Shanghai Ninth People’s Hospital, Shanghai Jiao Tong University School of Medicine, Shanghai, China

**Keywords:** Traditional medicine, *Rhodiola rosea*, *Ganoderma lucidum* spore, grape seeds, adverse effects

## Abstract

**Context:**

The traditional medicine 2036 Specialty-Qiangxin recipe (2036S-QXR) has been widely used in China to improve cardiac function, prevent stroke, and strengthen the immune system. However, its long-term toxicity remains unknown.

**Objective:**

The present study evaluates the long-term toxicity of 2036S-QXR in rats.

**Materials and methods:**

2036S-QXR (0.6, 1.2, and 2.4 g/kg body weight per day) was orally administered for 26 weeks to Wistar rats, while the rats in the control group received distilled water. The effects on urinary, hematological, biochemical, and histopathological parameters were investigated during the study period.

**Results:**

No significant changes in all tested parameters were observed in the 0.6 and 1.2 g/kg groups, compared with the control group (*p* < 0.05). Higher levels of alanine aminotransferase (46.00 ± 12.85 *vs.* 25.40 ± 3.36) and aspartate aminotransferase (152.40 ± 32.52 *vs.* 111.40 ± 18.78) were observed after 13 weeks in the female rats in the 2.4 g/kg group compared with the control group (*p* < 0.05), but these returned to the control levels after the recovery period (*p* > 0.05). Several cases displayed the presence of urine protein (3/7 males and 3/7 females) and mild lesions in the kidney (10/20) and thymus (5/20) in the 2.4 g/kg group, without significant changes compared with the control group (*p* > 0.05).

**Discussion and conclusions:**

The present study shows that 2036S-QXR does not cause long-term toxicity, supporting its therapeutic use. To further determine the optimal doses, future studies should test more doses and include more animals in each group.

## Introduction

Herb plants are traditionally used to treat various ailments worldwide, particularly in developing countries (Tilburt & Kaptchuk 2008; Oyebode et al. 2016). For some local communities, herb plants are the primary or only healthcare products available. They are usually considered safe and harmless, given that they are derived from natural sources (Bhat et al. 2019). However, the toxicity of herb plants has been underestimated, and there is inadequate information regarding the safety and toxicity risks associated with their use (Charen & Harbord 2020; Liu et al. 2020). It has been reported that herb plants may cause adverse effects, such as hepatotoxicity and nephrotoxicity when consumed repeatedly over a long period (van Quan et al. 2020; Xu et al. 2020). Thus, it is necessary to evaluate the toxicity profile of herb plants to provide recommendations regarding their safe use.

The Chinese medicine 2036 Specialty-Qiangxin recipe (2036S-QXR), consisting of three herb plant components, the *Rhodiola rosea* L. (Crassulaceae) extract, *Ganoderma lucidum* Karst (Ganodermataceae) spore powder, and grape seed proanthocyanidin extract, has been widely used as a health care product to improve cardiac function, prevent stroke, and strengthen the immune system. Our and other previous studies have shown the various therapeutic effects of *Ganoderma lucidum* spores (Zhang and Zeng 2004; Cheng et al. 2007; Gao et al. 2010; Zhou et al. 2012; Pan et al. 2019), the extract of *Rhodiola rosea* (Qu et al. 2009; Roumanille et al. 2020; Sun et al. 2020), and grape seed extract (Zhang et al. 2011; Huang et al. 2020; Eid et al. 2021). However, a systematic evaluation of the long-term toxicity of 2036S-QXR still remains unknown. Therefore, the present study was designed to determine the long-term toxicity of 2036S-QXR in Wistar rats.

## Materials and methods

### Experimental animals

Adult male and female Wistar rats (80–100 g, 4-weeks-old) were used in this study. The animals were obtained from the Charles River Laboratory, Beijing, China (License No. SCXK 2007–0001). Animals were kept under a controlled temperature (25 ± 2 °C), humidity (40–70%), and a 12 h light/dark cycle in the animal centre. A regular pellet diet and water were provided *ad libitum*. The rats were allowed to acclimatize for 5 d before the experimental procedures. All animal procedures were performed following the Guidelines for Animal Care and Use and were approved by the Institutional Animal Ethics Committee of Shenzhen University.

### Plant material

The 2036S-QXR capsules were obtained from Holistol International Co., Ltd., Hong Kong, China (License No. B071203R). 2036S-QXR consisted of 60% *Rhodiola rosea* hydroalcoholic extract, 38% *Ganoderma lucidum* spore powder, and 2% grape seed proanthocyanidin extract, all of which were obtained from Guangzhou Baoxing Bio-Technologies Co., Ltd., Guangdong, China. Briefly, the roots of *Rhodiola rosea* were dried and ground using a grinder. The coarse powder was then extracted twice using 70% alcohol for 2 h each time. The alcohol extract was condensed using a vacuum, collected via ethanol precipitation, and dried to produce a reddish-brown powder called the *Rhodiola rosea* extract. Raw *Ganoderma lucidum* spores were obtained by washing and drying the fungi, followed by grinding the spores into a powder. The grape seed proanthocyanidin was extracted using 50% alcohol and counter currently extracted with ethyl acetate. The grape seed proanthocyanidin extract was filtered and concentrated under a vacuum to produce the powder.

### Long-term toxicity study

Repeated dose toxicity studies were conducted according to the Organization of Economic Cooperation and Development (OECD) guidelines 407 (OECD 2008). The animals were randomly divided into four groups and were orally administered single daily doses of 0, 0.6, 1.2, and 2.4 g/kg of body weight of 2036S-QXR dissolved in distilled water. The control group (0 g/kg) and 2.4 g/kg group included 15 females and males each, while the 0.6 and 1.2 g/kg groups included ten females and males each. The treatment was continued for 26 weeks. Subsequently, in some animals, 2036S-QXR was replaced with an equal volume of distilled water. The animals were used to investigate the reversibility, persistence, or delayed occurrence of toxicity for four weeks (recovery period). All animals were provided with standard food and water *ad libitum* during the study period and were constantly monitored for signs of morbidity and mortality.

### Bodyweight and food intake

Bodyweight was measured once per week during the entire study period. Food intake was measured weekly and determined by subtracting the leftover food from the measured quantity provided in the previous week.

### Urinalysis

Urinalysis was performed at weeks 13 and 26 of the treatments and the end of the recovery period. The 24 h urine samples (from the morning after dosing to the morning on the day of urinalysis) were collected from the animals. The pH of the urine samples was measured, and the presence of occult blood, nitrite, urobilinogen, bilirubin, urine protein, glucose, and ketones in the urine samples was tested using a urinary test paper and were expressed as −, ±, +, or ++.

### Hematological and biochemical analyses

After the treatments for 13 and 26 weeks and at the end of the recovery period, the rats were anaesthetized using ketamine (40 mg/kg) and xylazine (5 mg/kg). Blood samples (2 mL from each rat) were collected from the retroorbital sinus into ethylenediaminetetraacetic acid tubes for hematological analyses and dry tubes for biochemical analyses. After blood sampling, the rats were euthanized by intraperitoneal injection of 150 mg/kg sodium pentobarbital. Hematological analyses were performed using an automatic hematological analyzer (Sysmex Corporation, Kobe, Japan). The hematological parameters measured included total red blood cell (RBC) count, total white blood cell (WBC) count, haemoglobin (HB) level, platelet (PLT) count, lymphocyte ratio, neutrophil ratio, and prothrombin time (PT). The blood samples for biochemical analyses were centrifuged at 1800 *g* for 15 min, and the sera were collected and stored at −20 °C. Biochemical parameters, including alanine aminotransferase (ALT), aspartate aminotransferase (AST), alkaline phosphatase (ALP), total bilirubin (TBIL), total protein (TP), albumin (ALB), cholesterol (CHOL), creatinine (CREA), and blood urea nitrogen (BUN) levels, were determined using a fully automatic biochemical analyzer (Beckman Counter Cx4 Pro; Beckman Coulter, Inc., Brea, CA, USA).

### Pathological examination

#### Gross necropsy

Following euthanasia with 150 mg/kg sodium pentobarbital, all animals were subjected to gross necropsy by an expert blinded to the treatment conditions, the body's external surface, all orifices, and the cranial, thoracic, and abdominal cavities, as well as their contents. The absolute organ weights of the heart, liver, spleen, lungs, kidneys, brain, thymus, adrenal glands, uterus, ovaries, and testes were measured immediately after dissection to avoid drying. The ratio of organ weight to body weight (relative organ weight) was then determined.

#### Histopathological analysis

The following organs and tissues were collected, fixed in 10% neutral buffered formalin, and embedded in paraffin: heart, liver, spleen, lungs, kidneys, brain, thymus, adrenal glands, uterus, ovaries, testes, parathyroid glands, stomach, duodenum, jejunum, ileum, pancreas, epididymis, urinary bladder, and pituitary gland. The paraffin-embedded sections were stained with haematoxylin and eosin. Histopathological changes were examined under a light microscope (Olympus CH02; Olympus Corporation, Tokyo, Japan).

#### Statistical analysis

GraphPad Prism version 8 (GraphPad Software, Inc., La Jolla, USA) was used for the data analysis. The results of body weight and food intake are presented as the mean ± standard deviations (mean ± SD) and were analyzed using a two-way repeated-measures analysis of variance (ANOVA). The data of the hematological values, biochemical values, and relative organ weight are presented as the mean ± SD, and the differences between each treatment group and the control group were compared using a one-way ANOVA followed by Dunnett’s test; Differences with *p-*values <0.05 were considered to be statistically significant. Histopathological changes were analyzed using Fisher’s exact test. Urinary parameters were analyzed using Fisher’s exact test (between the number of ‘−’ cases and the total number of ‘±’, ‘+’, and ‘+ +’ cases).

## Results

### General observation and mortality

No deaths were observed in any group throughout the study period. Moreover, no abnormal changes in general behaviour or other physiological activities were observed.

### Bodyweight and food intake

**Figure 1.**
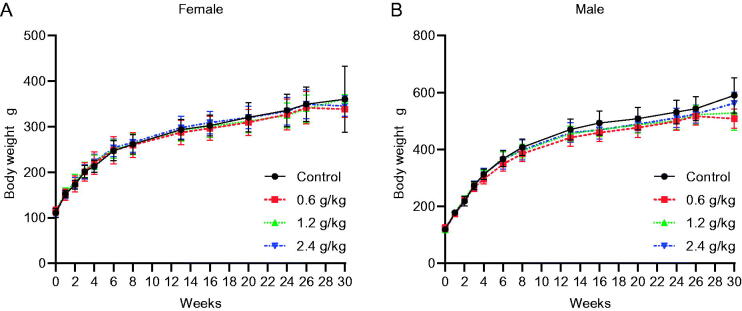
Effects of oral 2036S-QXR on body weight during the 26-week period of administration and at the end of the 4-week recovery period. (A) Changes in body weight in female rats. (B) Changes in body weight in male rats. The number of female and male animals in each group (0–13 weeks) was 15. The number of female and male animals in each group (13–26 weeks) was 10. The number of female and male animals in each group at the end of the 4-week recovery period (30 weeks) was 3. There were no significant differences in the bodyweight of males and females in the treatment groups compared with the control group (*p* > 0.05). Mean ± SD.

The body weight steadily increased in the male and female rats in all groups. No treatment-related effects were observed during the study period (Figure 1). The food intake was also not significantly different between each treatment group and the control group during the study period (Table 1).

**Table 1.** Food intake (g/100g body weight/week) after the administration of 2036S-QXR.

Sexes	Time (weeks)	Mouse number	Control	0.6 g/kg	1.2 g/kg	2.4 g/kg
Female						
	0	15	126.1	129.8	117.9	127.1
	1	15	118.7	131.6	108.5	124.3
	2	15	111.6	119.3	101.7	107.1
	3	15	109.8	106.2	103.5	104.9
	4	15	98.3	97.7	96.8	93.6
	6	15	83.4	79.4	77.8	83.5
	8	15	76.1	73.4	66.3	79.7
	13	15	68.1	59.8	56.9	72.2
	16	10	63.8	59.7	53.7	62.8
	20	10	54.9	57.1	52.9	57.7
	24	10	53.3	54.2	51.7	56.1
	26	10	54.7	52.8	55.5	54.6
	Recovery period	3	42.8	45.5	44.9	45.3
Male						
	0	15	157.0	151.4	168.5	159.5
	1	15	122.4	121.6	122.0	125.3
	2	15	107.6	106.3	104.8	111.0
	3	15	91.8	94.2	94.2	94.6
	4	15	85.1	84.9	83.6	89.3
	6	15	64.5	64.6	65.8	65.9
	8	15	60.3	59.6	58.3	62.6
	13	15	50.2	49.3	49.9	52.5
	16	10	44.6	41.9	42.0	41.7
	20	10	42.8	42.4	41.2	43.7
	24	10	40.6	40.0	40.8	42.0
	26	10	42.4	43.9	44.4	44.9
	Recovery period	3	34.5	33.7	32.6	34.7

*Note*. Data represents the total food intake of all the mice in each group per week relative to their body weights. The *p*-values in all treatment groups were not significant compared with the control group (0 g/kg).

### Urinary parameters

The results of the urinary analysis are summarized in Table 2. After 13 weeks of administration, no significant differences in urinary pH values were observed in any group. Furthermore, urinary occult blood, nitrite, urobilinogen, bilirubin, protein, glucose, and ketones were negative in all groups at this time point. After 26 weeks of administration, the detection of urine protein was positive in three males (*n* = 7) and three females (*n* = 7) in the 2.4 g/kg group; however, the differences were not significant compared with the control group, as determined using Fisher’s exact test (*p* > 0.05). Similarly, animals in the 0.6 and 1.2 g/kg groups showed no significant changes in urinary protein compared with those observed in the control group (*p* > 0.05). The remaining parameters were negative in all groups. At the end of the recovery period, no significant differences in pH were observed (*p* > 0.05), and the other urinary parameters, including urine protein, were negative in all groups. The slight increase in urine protein observed in the 2.4 g/kg group suggested that the long-term administration of 2.4 g/kg 2036S-QXR may cause mild nephrotoxicity, which can be reversed after the withdrawal of 2036S-QXR.

**Table 2.** Urinary analysis after the administration of 2036S-QXR.

Times	Sexes	Doses	pH	Occult blood				Nitrite				Urobilinogen				Bilirubin				Protein				Glucose				Ketones			
				−	±	+	++	−	±	+	++	−	±	+	++	−	±	+	++	−	±	+	++	−	±	+	++	−	±	+	++
13 weeks	Female	Control	6.00 ± 0.00	5				5				5				5				5				5				5			
		2.4 g/kg	6.40 ± 0.55	5				5				5				5				5				5				5			
	Male	Control	6.00 ± 0.00	5				5				5				5				5				5				5			
		2.4 g/kg	6.00 ± 0.00	5				5				5				5				5				5				5			
26 weeks	Female	Control	6.14 ± 0.38	7				7				7				7				5	1	1		7				7			
		0.6 g/kg	6.71 ± 0.76	7				7				7				7				6	1			7				7			
		1.2 g/kg	6.57 ± 0.53	7				7				7				7				6	1			7				7			
		2.4 g/kg	6.28 ± 0.76	7				7				7				7				4		2	1	7				7			
	Male	Control	6.28 ± 0.49	7				7				7				7				5	1	1		7				7			
		0.6 g/kg	6.14 ± 0.38	7				7				7				7				6	1			7				7			
		1.2 g/kg	6.42 ± 0.53	7				7				7				7				6	1			7				7			
		2.4 g/kg	6.42 ± 0.53	7				7				7				7				4		2	1	7				7			
Recovery period	Female	Control	6.00 ± 0.00	3				3				3				3				3				3				3			
		0.6 g/kg	6.00 ± 0.00	3				3				3				3				3				3				3			
		1.2 g/kg	5.67 ± 0.58	3				3				3				3				3				3				3			
		2.4 g/kg	6.33 ± 0.58	3				3				3				3				3				3				3			
	Male	Control	6.00 ± 0.00	3				3				3				3				3				3				3			
		0.6 g/kg	6.00 ± 0.00	3				3				3				3				3				3				3			
		1.2 g/kg	6.33 ± 0.58	3				3				3				3				3				3				3			
		2.4 g/kg	6.33 ± 0.58	3				3				3				3				3				3				3			

*Note*. pH value is mean ± SD. The *p*-values in all treatment groups were not significant compared with the control group using Fisher’s exact test.

### Hematological parameters

**Figure 2.**
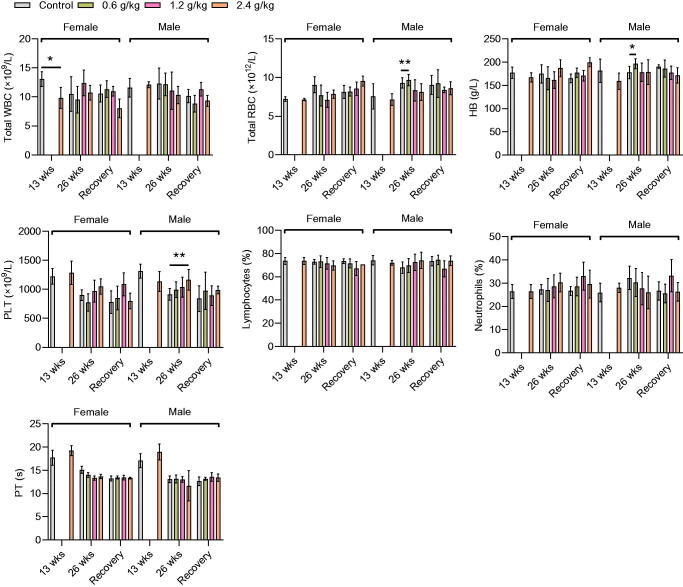
Hematological parameters of the rats after administration of 2036S-QXR for 13 and 26 weeks, and at the end of the recovery period. *p < 0.05 and **p < 0.01 as compared to the control group (0 g/kg). Mean ± SD. *n* = 5 for 13 weeks, *n* = 7 for 26 weeks, and *n* = 3 for the recovery period.

The effects of repeated oral administration of 2036S-QXR on hematological parameters are shown in Figure 2. Administration of 2036S-QXR at a dose of 2.4 g/kg for 13 weeks caused a slight decrease in the WBC count (*p* < 0.05) in female rats, but not in male rats, compared with the control group (Table 3). No significant differences in RBC count, HB level, PLT count, Lymphocytes, Neutrophils, and PT were observed between the 2.4 g/kg and control groups (*p* > 0.05). After 26 weeks of administration, 0.6 g/kg 2036S-QXR caused a slight increase in RBC count and HB level in male rats compared with the control male rats (*p* < 0.01 and *p* < 0.05, respectively), but not in female rats. Moreover, male rats in the 2.4 g/kg group exhibited an increase in PLT count compared with the control male rats after 26 weeks of administration (*p* < 0.01). All other hematological parameters were not significantly altered in the treatment groups compared to those in the control group (*p* > 0.05). At the end of the recovery period, animals in the treatment groups displayed no significant changes in any hematological parameters compared with the control group (*p* > 0.05). Since the slight changes in WBC count, RBC count, and HB level were not dose-dependent, they were suggested to be of non-toxicological significance. Therefore, these results indicate that the long-term administration of 2036S-QXR may not cause apparent hematological toxicity.

**Table 3.** Incidence of histopathological lesions in various organs after the administration of 2036S-QXR for 26 weeks and at the end of the recovery period.

Organs	Microscopic findings	Control	0.6 g/kg	1.2 g/kg	2.4 g/ml
Heart	Normal	20/20	20/20	20/20	20/20
Liver	Cell infiltration (slight)	10/20	10/20	6/20	9/20
Lung	Interstitial pneumonia (slight); cell infiltration (slight)	11/20	11/20	11/20	7/20
Spleen	Normal	20/20	20/20	20/20	20/20
Kidney	Basophilic lesions; Scattered interstitial nephritis; dilated tubules; protein casts (slight)	9/20	7/20	6/20	10/20
Bladder	Normal	20/20	20/20	20/20	20/20
Adrenal	Normal	20/20	20/20	20/20	20/20
Stomach	Normal	20/20	20/20	20/20	20/20
Duodenum	Normal	20/20	20/20	20/20	20/20
Jejunum	Normal	20/20	20/20	20/20	20/20
Ileum	Normal	20/20	20/20	20/20	20/20
Uterus	Normal	10/10	10/10	10/10	10/10
Ovary	Normal	10/10	10/10	10/10	10/10
Testis	Accumulation of macrophages (slight)	0/10	0/10	2/10	0/10
Epididymis	Normal	10/10	10/10	10/10	10/10
Prostate	Chronic prostatitis (slight)	3/10	1/10	2/10	2/10
Cerebrum	Normal	20/20	20/20	20/20	20/20
Cerebellum	Normal	20/20	20/20	20/20	20/20
Pituitary	Normal	20/20	20/20	20/20	20/20
Thymus	Thymus atrophy; vesicles (slight)	2/20	2/20	4/20	5/20
Pancreas	Normal	20/20	20/20	20/20	20/20
Thyroid	Normal	20/20	20/20	20/20	20/20
Parathyroid	Normal	20/20	20/20	20/20	20/20

*Note*. Data are *n*/*N*, *n* represents the animal number that has the histopathological finding and *N* represents the total animal number examined.

### Biochemical parameters

**Figure 3.**
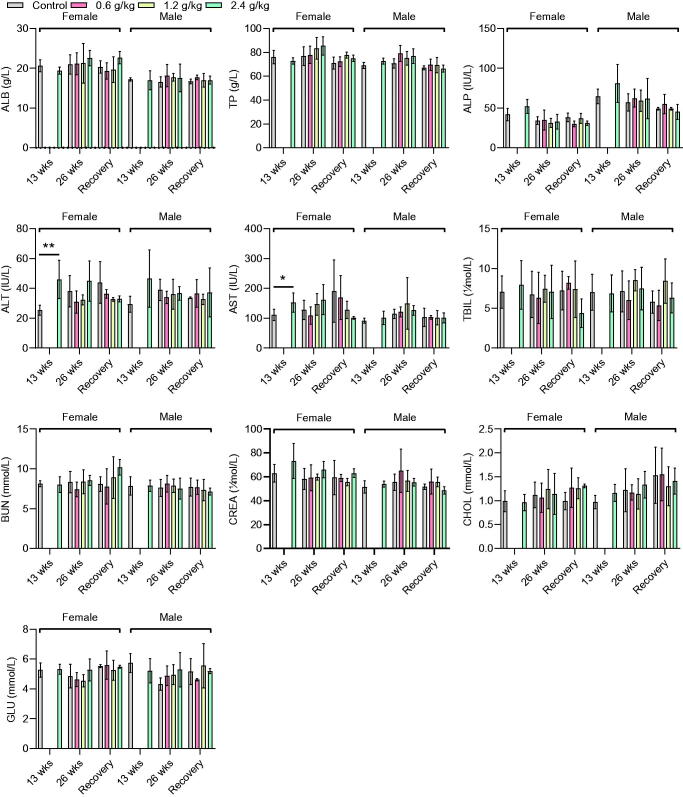
Biochemical parameters of rats after administration of 2036S-QXR for 13 and 26 weeks, and at the end of the recovery period. *p < 0.05 and **p < 0.01 as compared to the control group (0 g/kg). Mean ± SD. *n* = 5 for 13 weeks, *n* = 7 for 26 weeks, and *n* = 3 for the recovery period.

An increase in the levels of ALT (46.00 ± 12.85 IU *vs.* 25.40 ± 3.36 IU, *p* < 0.01) and AST (152.40 ± 32.52 IU/L *vs.* 111.40 ± 18.78 IU/L, *p* < 0.05) was observed in female rats in the 2.4 g/kg group after 13 weeks of administration, compared with the female rats in the control group (Figure 3). However, no significant changes in the other biochemical parameters were observed in female and male rats in the 2.4 g/kg group, compared with those in the control group (*p* > 0.05, Figure 3). After 26 weeks of administration, although the female rats in the 2.4 g/kg 2036S-QXR group appeared to have higher ALT and AST levels than the control group, this difference was not significant (*p >* 0.05). Furthermore, no significant alterations in other biochemical parameters were observed between the rats from the treatment and control groups (*p* > 0.05). At the end of the recovery period, none of the biochemical values in the treatment groups exhibited significant changes compared with the control group (*p* > 0.05). The changes in ALT and AST levels collectively indicated that long-term administration of 2.4 g/kg 2036S-QXR may be associated with mild hepatotoxicity.

### Gross necropsy

**Figure 4.**
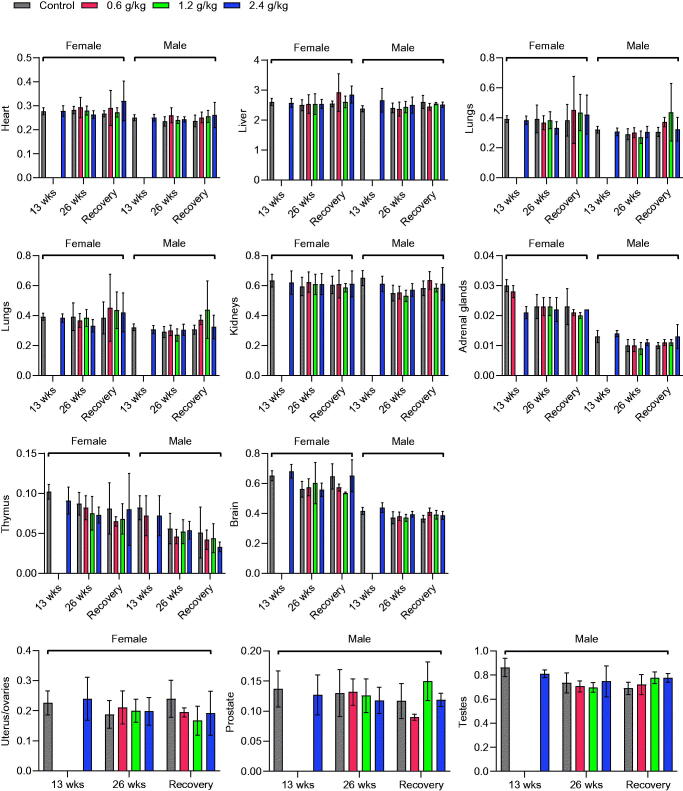
Effects of oral 2036S-QXR on the relative organ weight (g/100 g body weight). The *p*-values in all treatment groups were not significant compared with the control group. Mean ± SD. *n* = 5 for 13 weeks, *n* = 7 for 26 weeks, and *n* = 3 for the recovery period.

Gross examination of organs and body cavities revealed no gross lesions or other abnormalities after 13 and 26 weeks of 2036S-QXR administration at doses of 0.6, 1.2, and 2.4 g/kg and at the end of the recovery period. In addition, the measurement of organ weight revealed no significant differences between the rats from each treatment group and the control group during the experimental period (*p* > 0.05, Figure 4).

### Pathological examination

**Figure 5.**
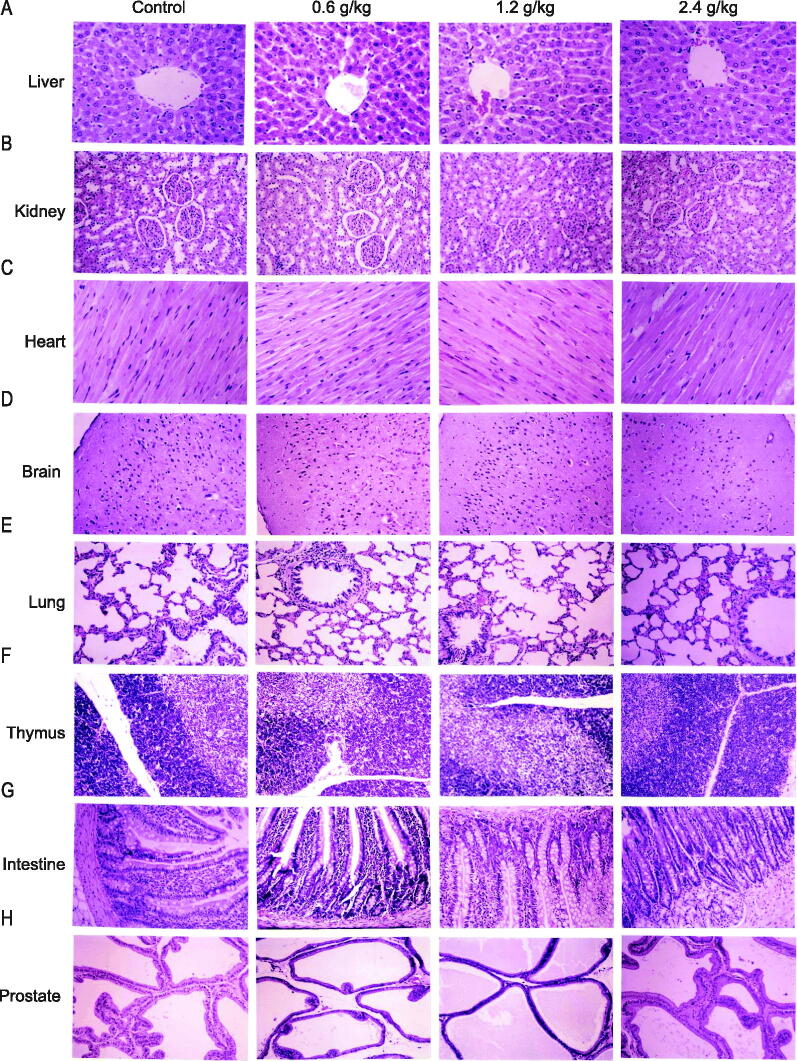
Histopathological organ examination after administration of 2036S-QXR using haematoxylin and eosin staining (10×). (A) Representative images of liver sections. (B) Representative images of kidney sections. (C) Representative images of heart sections. (D) Representative images of brain sections. (E) Representative images of lung sections. (F) Representative images of thymus sections. (G) Representative images of intestinal sections. (H) Representative images of prostate sections.

The histopathological findings of the organs after the administration of 2036S-QXR for 26 weeks and at the end of the recovery period are described in Table 3 and Figure 5. The heart, spleen, bladder, adrenal gland, stomach, duodenum, jejunum, ileum, uterus, ovary, epididymis, cerebrum, cerebellum, pituitary gland, pancreas, thyroid gland, and parathyroid gland exhibited normal histology in all groups. Slight histopathological changes were observed in the liver, lungs, testes, and prostate of rats from all groups, but no significant differences in the frequency of these changes were observed in the treatment groups, compared with the control group (*p* > 0.05). Thus, these pathological changes were considered spontaneous or incidental.

The kidney sections displayed basophilic lesions, scattered interstitial nephritis, dilated tubules, and protein casts in the distal convoluted tubules of the renal cortex and medulla in samples from both the treatment and control groups (Table 3 and Figure 5(B)). Since the pathological renal changes identified in the treatment groups exhibited no significant differences in the frequency, degree, and lesion area compared with those in the samples from the control group, these pathological alterations may be attributed to increasing age and may not have been caused by the treatment. Nevertheless, kidney function should be monitored during the long-term administration of high-dose 2036S-QXR when considering the high frequency of renal lesions in the 2.4 g/kg 2036S-QXR group (10/20).

Tissue atrophy and vesicles were observed in the thymus glands of the rats from the treatment and control groups (Table 3 and Figure 5(F)). Although the 1.2 and 2.4 g/kg 2036S-QXR groups had more cases showing pathologies than the control group (4/20, 5/20, and 2/20, respectively), the differences in the frequency of pathologies were not significant (as detected using a Fisher’s exact test), and no notable differences in the degree and area of atrophy were identified between the animals from the treatment groups and the control group. Thus, the pathological findings of the thymus were considered to have no toxicological significance.

## Discussion

Herb plants have gained increasing popularity in developing countries. Toxicity assessment is essential for the safety of herb plants, as toxicity is a concern to the medical community and the public. The Chinese medicine 2036S-QXR has long been used to improve cardiac function and prevent stroke. The present study investigated the long-term toxicity of 2036S-QXR in rats and identified that the administration of 1.2 g/kg 2036S-QXR for 26 weeks was not associated with toxicity, as evidenced by a lack of hazardous effects on general health and the function of vital organs. However, administration of 2036S-QXR at a dose of >2.4 g/kg may be associated with mild hepatotoxicity and nephrotoxicity in rats. Therefore, it is recommended that the liver and kidney functions be monitored during the long-term clinical use of a high dose of 2036S-QXR. The findings of the present study may provide a guide for selecting a safe dose of 2036S-QXR for further human use.

The recommended clinical dose of the 2036S-QXR capsule was 30 mg/kg/day. Therefore, in the current study, the doses of 2036S-QXR in the long-term toxicity test were set as 0.6, 1.2, and 2.4 g/kg, which corresponded to 20, 40, and 80 times the clinically recommended dose, respectively. Administration of 2036S-QXR at any of the tested doses for 26 weeks did not affect the general behaviour or survival of animals, and no deaths were observed during the treatment period, thereby confirming the safety of 2036S-QXR at the tested doses. In addition, 2036S-QXR administration did not produce significant differences in body weight or food intake compared with the control group, suggesting that 2036S-QXR did not alter appetite. A change in body weight is considered a valuable indicator of the adverse effects of drugs or phytomedicines (OECD 2001). Moreover, the measurement of food intake is another important factor for evaluating the safety of a therapeutic agent, as the intake of nutrients is fundamental for maintaining the physiological status when receiving a drug test, and inadequate nutritional intake may cause inaccurate responses (Alli et al. 2015).

In the current urinary analyses, it was demonstrated that more female and male rats showed positive results for urine protein in the 2.4 g/kg 2036S-QXR group than in the control group, but this was not statistically significant. The determination of BUN and CREA levels in the biochemical analyses was used to examine renal secretory function. In the present study, no significant differences in their levels were observed between the treated and control rats, suggesting that the long-term administration of 2036S-QXR did not adversely affect renal secretory function. Although there were no significant differences in the pathological changes in the kidneys, such as basophilic lesions, scattered interstitial nephritis, dilated tubules, or protein casts, in the rats from the treatment groups compared with those from the control group, the presence of pathological lesions and increased urine protein levels after the long-term administration of 2.4 g/kg 2036S-QXR indicated that the long-term oral administration of 2036S-QXR at this high dose may exacerbate the spontaneous pathological changes in the kidneys. Thus, the long-term use of 1.2 g/kg 2036S-QXR is relatively safer. Since the kidney is the most vulnerable to herb plants (Xu et al. 2020), it is necessary to take precautions to prevent nephrotoxicity during the long-term administration of 2036S-QXR.

Analysis of hematological parameters is a good predictor of the toxic potential of drugs, as blood cells are the first cells to be exposed to drug toxicity (Barbosa et al. 2016). The results of the present study demonstrated that the administration of 0.6 g/kg 2036S-QXR for 26 weeks increased the RBC count and HB level in the treated male rats compared with the control male rats. Furthermore, a significant increase in the PLT counts was observed in male rats administered 2.4 g/kg 2036S-QXR for 26 weeks, compared to the control male rats. However, the increases in RBC count, HB level, and PLT count were reversed at the end of the recovery period. In addition, their alterations were sex-related and dose-independent, suggesting that these alterations were likely not the result of hematological toxicity induced by the administration of 2036S-QXR.

Enzymes are implicated in almost all metabolic activities of the body; thus, the determination of the activities of these enzymes in body tissues and fluids is a valuable tool for evaluating the toxicity of plant extracts to tissues (Zhang et al. 2018; Dandashire et al. 2019). Enzymes, such as ALT and AST are generally used to assess liver function. In our study, administration of 2.4 g/kg 2036S-QXR for 13 weeks increased the levels of ALT and AST in female rats compared with the female control rats. After 26 weeks of administration, rats in the 2.4 g/kg 2036S-QXR group still appeared to have higher ALT and AST levels than those in the control group, but there were no significant differences. In contrast, 0.6 and 1.2 g/kg 2036S-QXR administration for 26 weeks did not induce an increase in ALT and AST levels compared with the control group. Moreover, there were no significant differences between the ALT and AST levels in the rats from the treatment and control groups at the end of the recovery period. It was found that the total protein and ALB levels in serum were not significantly altered at any of the tested doses compared with the control group, suggesting that different doses of 2036S-QXR did not impair the synthetic function of the liver. Furthermore, 2036S-QXR administration did not induce a significant change in bilirubin levels in the rats from the treatment groups at any dose compared with those from the control group. No significant pathological changes were observed in the gross liver and histopathological sections between the treatment and control groups. The long-term administration of 2.4 g/kg 2036S-QXR may only produce mild hepatotoxicity, which could be eliminated after the withdrawal of the treatment.

In this study, the relative organ weight and histopathological examination revealed no significant changes in most organs after long-term administration of 2036S-QXR. It was suggested that the mild degree of histopathological alterations in the liver, kidney, testis, prostate, and thymus in all groups might be spontaneous, incidental, or age-related, with no toxicological significance.

Our study provided a comprehensive evaluation on the long-term toxicity of 2036S-QXR in rats and found that long-term oral administration of 2036S-QXR at doses of 0.6, 1.2, and 2.4 g/kg was relatively safe in rats. Nonetheless, oral administration of a high dose (2.4 g/kg) of 2036S-QXR over a long period should be cautiously monitored, as it may cause mild hepatotoxicity and nephrotoxicity. Based on the current results, the no-observed-adverse-effect level for 2036S-QXR in rats was set as 1.2 g/kg per day, corresponding to 40 times the clinically recommended dose. Additionally, it is recommended that kidney and liver function be monitored during the long-term administration of high 2036S-QXR doses.

## Data Availability

The authors confirm that the data supporting the findings of this study are available within the article.
